# Platelet-Rich Fibrin in MRONJ Management: A Prospective Comparative Study on Its Effectiveness in Prevention and Treatment

**DOI:** 10.3390/medicina61040625

**Published:** 2025-03-28

**Authors:** Raluca Maracineanu, Anca Tudor, Ivona Hum, Florin Urtila, Felicia Streian, Serban Talpos-Niculescu, Marilena Motoc

**Affiliations:** 1 Doctoral School, “Victor Babeș” University of Medicine and Pharmacy, 300062 Timisoara, Romania; raluca.zibileanu@umft.ro (R.M.); ivona.ursu@umft.ro (I.H.); 2 Department of Functional Sciences, “Victor Babeș” University of Medicine and Pharmacy, 300041 Timisoara, Romania; atudor@umft.ro; 3 Discipline of Oral and Maxillofacial Surgery, Faculty of Dental Medicine, “Victor Babeș” University of Medicine and Pharmacy, 300062 Timisoara, Romania; urtila.florin@umft.ro (F.U.); streian.felicia@umft.ro (F.S.); 4 Department of Biochemistry and Pharmacology, Discipline of Biochemistry, “Victor Babeș” University of Medicine and Pharmacy, 300041 Timisoara, Romania; motoc.marilena@umft.ro

**Keywords:** MRONJ, platelet-rich fibrin, primary suture, normal healing, non-healing, bisphosphonates, denosumab, antiresorptive and antiangiogenic medication

## Abstract

*Background and Objectives*: Medication-related osteonecrosis of the jaw (MRONJ) was first recognized as a disease entity and reported in the literature in 2003. Within a few years, the incidence of MRONJ has increased significantly, to the point where now it can be seen in every dental clinic around the world. Its prevention and management still remain major challenges for dentists and oral and maxillofacial surgeons. *Materials and Materials and Methods*: This prospective clinical study was conducted at the Oral and Maxillofacial Surgery Clinic in Timisoara for a 6-month period and included a total of 85 patients under chronic antiresorptive and antiangiogenic medication. There were two groups of patients: G1 received PRF growth factors, while the other group, G2, was treated with classical surgical methods. Post-operative wound healing was assessed at 2, 4, and 8 weeks by monitoring the absence of local gingival dehiscence, suprainfection, or loco-regional fistulas, both in cases of dental extractions and sequestrectomies in MRONJ cases. *Results:* The use of PRF in post-extraction sockets in patients predisposed to developing MRONJ aids in local healing in 96% of cases, compared to cases where it was not used, in which normal healing occurred in only 64.29% of patients; there was a significant difference between the two groups (*p* = 0.016). In MRONJ confirmed cases, application of PRF after excisional debridement of necrotic bone does not appear to have the same therapeutic value as in post-extractional sockets, with a *p*-value of 0.299 indicating no statistical significance. *Conclusions*: PRF use can be considered an effective approach in preventing osteonecrotic complications following dental extractions in patients with antiresorptive treatment. Additional studies are needed to establish its role in MRONJ confirmed cases.

## 1. Introduction

Medication-related osteonecrosis of the jaw (MRONJ) is one of the most widely discussed current topics in oral and maxillofacial surgery. First described in the literature by R.E. Marx in 2003 as “a growing epidemic”, it has significantly increased in prevalence in recent years [1]. Both the prevention and treatment of this condition are of great interdisciplinary interest, especially given the increasing number of patients on chronic antiangiogenic and antiresorptive medication such as bisphosphonates or denosumab [2,3].

According to the 2022 update of the AAOMS Position Paper, three essential criteria must be met simultaneously to define a case of osteonecrosis of the jaws as MRONJ: chronic treatment with antiresorptive or antiangiogenic medication, the absence of a history of tumor or metastasis in the jawbones or prior radiotherapy to that area, and the presence of an oral dehiscence with or without a fistula persisting for more than 8 weeks [4].

Platelet-rich fibrin (PRF), developed by J. Choukroun et al., is an autologous product obtained, without the use of anticoagulants, from the patient’s peripheral blood [5]. It contains high concentrations of various platelet growth factors kept in a natural fibrin matrix with prolonged local release. It helps in reducing post-operative local edema and pain and accelerates local healing by inducing neovascularization and simultaneously lowering the risk of bacterial contamination. These beneficial effects on the local healing mechanism are supported by four fundamental mechanisms: activation of angiogenesis, circulating stem cell trapping, favorable immune control, and promotion of local epithelialization at the wound margins [6].

In oral tissue wounds, the application of PRF prevents gingival dehiscence by promoting the healing of both soft and hard tissues. A systematic review from 2021 shows that the application of PRF in post-extraction sockets significantly improves local tissue healing one week after extractions. Additionally, in the first 8 weeks, in cases where PRF was applied, bone loss was significantly lower compared to spontaneous healing [7].

Many recent studies show that the mechanism of action and biological properties of PRF-type tissue growth factors can aid both in the prevention of MRONJ development and in its surgical treatment. The bone and soft tissue healing process is stimulated by the application of growth factors that act directly on tissue regeneration by accelerating mucosal wound healing, reducing tissue inflammation, and improving local vascularization [8,9,10,11,12,13,14,15]. The present study brings new and important information to the current body of literature regarding the effectiveness of PRF application as a method of prevention and/or treatment of MRONJ, compared to the classical methods used. Although there are currently many studies highlighting the overwhelming importance of using PRF in the treatment and prevention of MRONJ, a clear comparison between this adjuvant method and traditional surgical techniques using primary closure is lacking. Moreover, there are no definitive studies demonstrating whether PRF is also effective in different stages of already confirmed MRONJ, where curettage and sequestrectomy procedures result in significant bone defects, carrying a high risk of local superinfection and recurrence.

The aim of this study is to evaluate the importance of PRF application both preventively, in post-extraction sockets of patients undergoing chronic antiangiogenic and antiresorptive treatment to reduce the risk of MRONJ development and bone exposure, and as an adjunctive method in the surgical treatment of confirmed MRONJ cases to reduce the local gingival dehiscence.

## 2. Materials and Methods

This research constitutes a prospective controlled clinical study conducted in adherence to the guidelines outlined in the Helsinki Declaration of 1975. The investigation spanned a duration of 6 months and was carried out in the Clinical Municipal Emergency Hospital of the Discipline of Oral and Maxillofacial Surgery, “Victor Babeș” University of Medicine and Pharmacy, Timișoara.

Ethical approval was secured from the Independent Ethics Committee of the University of Medicine and Pharmacy “Victor Babeș”, Timișoara, Romania (nr.51/2 October 2023).

To conduct this investigation, we selected all individuals undergoing chronic antiresorptive or antiangiogenic medication who sought care at the Oral and Maxillofacial Surgery Clinic in Timișoara between October 2023 and April 2024.

Patients were selected based on specific inclusion criteria: all subjects were under chronic medication with bisphosphonates or other antiresorptive drugs administered orally or parenterally for at least 1 year; patients required one or more dental extractions or were already diagnosed with MRONJ and were scheduled for sequestrectomy surgical intervention.

The exclusion criteria ruled out patients who had undergone head and neck radiotherapy sessions in the last 24 months, patients with tumor pathology or metastases in the cervicofacial region, patients with osteomyelitis of the maxillary bones or recent fractures, pregnant or breast-feeding women, and patients who had undergone other oral or dental surgical treatments in the last 3 months.

A total of 91 patients were selected based on the aforementioned criteria to participate in the study. Of these, a total of 6 patients were excluded from the study: 4 patients did not attend follow-up appointments, and the other 2 patients resumed bisphosphonate treatment during the healing period due to associated metastatic pathology.

Out of the remaining 85 patients in the study, after explanation of the protocol, 29 of them declined to have blood drawn for the application of autologous PRF material and were treated in the traditional way. The reasons mentioned by them included the extended working time, fear of needles, or apprehension about potential complications due to the new surgical protocol. As a result, two study groups were formed: Group 1 (G1), consisting of 56 patients who were treated with PRF application, and Group 2 (G2), consisting of 29 patients who were treated traditionally without local PRF application.

Both groups included high-risk patients without MRONJ who underwent dental extractions, as well as patients already diagnosed with MRONJ who received surgical treatment for existing lesions.

Tooth extractions were performed on a total of 39 patients categorized as high-risk for developing MRONJ; of these, 25 patients received PRF and were included in Group 1, while 14 did not and were included in Group 2. Patients who were already diagnosed with MRONJ before the procedure and underwent the treatment for the existing lesions totaled 46; they were divided into 31 patients in Group 1 (treated with PRF) and 15 patients in Group 2 (treated with traditional methods).

### 2.1. Statistical Assessment

JASPv0.18.3 software was applied for statistical analyses. Qualitative variables were described as percentages, while quantitative variables as mean ± standard deviation (SD) and median (interquartile interval). To compare independent series of numerical values, the Mann–Whitney U test was used for two series of numerical values, and the Kruskal–Wallis test was used in the case of more than two independent series of numerical values. The Chi-squared Fisher exact test was used to associate categorical data. A significance level of 0.05 was chosen for all tests. A G*Power test was used for contingency tables, with 80% power, a 0.05 level of significance, and 0.31 as an effect size.

### 2.2. Technical Procedures

Before any surgical procedures, all patients underwent imaging investigations such as orthopantomographs or dental CT scans. Additionally, a detailed medical history was obtained, focusing on the type of antiresorptive medication used, the mode of administration, the underlying condition for which it was prescribed, and any associated pathologies or medications that could affect healing, such as diabetes or the use of anticoagulant medication. The standard protocol followed in both G1 and G2 involved discontinuing antiresorptive treatment three months before the surgical intervention and maintaining this pause for an additional month post-operation. One day before the surgical procedure, patients began prophylactic antibiotic treatment with amoxicillin and clavulanic acid (1 g every 12 h) combined with metronidazole (250 mg every 12 h) and performed oral rinses twice a day with alcohol-free chlorhexidine 0.2% antiseptic solutions. This protocol was also applied for an additional 6 days post-operation. Patients allergic to amoxicillin were prescribed clindamycin (600 mg every 8 h).

All surgical interventions were performed by a single surgeon in order to avoid procedural errors or variations in technique that could modify the final results of the present study.

Local anesthesia was performed using articaine hydrochloride with epinephrine at a concentration of 1:100,000 or 1:200,000 depending on the patient’s associated general pathology and the desired hemostatic effect.

Dental extractions were performed as atraumatically as possible, followed by alveolar curettage. For patients in Group 1, PRF obtained from peripheral venous blood was applied in the post-extractional socket, followed by a cross-stitch suture with non-absorbable 4-0 polyamide thread. Patients in Group 2 received a simple intra-alveolar hemostatic material post-extraction, followed by a cross-stitch suture with the same type of 4-0 polyamide thread. In both groups, only a simple supra-alveolar suture was used, without creating a local gingival flap for a hermetic closure of the post-extraction area. All patients provided informed consent and were made aware of the potential complications associated with undergoing dental extraction while on medication.

For patients confirmed with MRONJ, debridement of granulation tissue and necrotic bone was performed. Bone sequestra were completely removed until healthy bone tissue was reached using elevators or bone forceps, without using bone drills or other rotary instruments to minimize the risk of bone necrosis. Local rinses were performed with antiseptic solutions containing 0.2% chlorhexidine and 0.9% sodium chloride. In the resulting bone defect, PRF membranes were applied for patients in G1, while hemostatic materials were applied for patients in G2. A mucoperiosteal flap was created, relaxed, and freely tensioned over the defect. The flap was tightly sutured with non-absorbable 4-0 polyamide thread.

To make the PRF, venous blood from the patient was drawn into 10 mL glass tubes with red caps, without the addition of anticoagulants. Following collection, the blood was promptly centrifuged at 1500 rpm for 8 min. Subsequently, the PRF clots were carefully extracted from the tubes and separated from the red blood cells. These PRF clots were then placed into containers and compressed to form PRF membranes. The quantity of PRF used was in accordance with the number of extractions or the size of the resulting alveolar defect after sequestrectomy.

Patients were provided with clear explanations of all standard post-extraction indications, such as strict oral hygiene, soft diet, and avoidance of local-region trauma to the operated area. Sutures were removed at 14 days post-operation, with follow-up on the progress of post-extraction alveolar socket closure or post-operative wound healing (in confirmed osteonecrosis cases) at 2, 4, and 8 weeks. The gingival healing indices monitored in the healing progression were represented by the presence or absence of the following: gingival dehiscence, local endo-oral or cervicofacial fistula, or local suprainfection indicated by the presence of purulent discharge. Therefore, cases were considered “normal healing” if the above-mentioned indices were absent at the 2-week follow-up. “Delayed healing” was defined as cases where one or more of the healing indices were initially present but gradually resolved between 4 and 8 weeks with the aid of daily oral rinses and strict hygiene accompanied by appropriate antibiotic treatment. The term “non-healing cases” was applied to situations where one or more gingival healing indices persisted beyond 8 weeks.

In accordance with the American Association of Oral and Maxillofacial Surgeons’ Position Paper on Medication-Related Osteonecrosis of the Jaws—2022 Update, a case of medication-related maxillary osteonecrosis (MRONJ) is defined by the presence of a persistent oral dehiscence lasting more than 8 weeks. All cases that achieve good gingival healing without oral dehiscence at 8 weeks after the surgery are considered resolved cases at that time [4]. Based on this case definition and in accordance with other similar studies in the literature, we decided to follow the local tissue healing over a period of 8 weeks.

Out of the 91 patients initially included in the study, four did not attend the 8-week follow-up and were therefore excluded from the study. The remaining 85 patients were monitored at 2, 4, and 8 weeks. After this period, periodic follow-ups were performed to monitor the evolution of the cases at 3, 6, and 12 months with no patients lost, but these data are not the subject of the current study. In the present study, we only monitored local gingival healing through the application or not of PRF, for which a follow-up period of 8 weeks is sufficient.

## 3. Results

Out of the total 91 patients initially included in the study, six patients were excluded for various reasons previously mentioned. This left a total of 85 patients, of which 68.23% were women and 31.77% men. Of these patients, 65.88% (G1) were treated with the application of PRF, while the remaining 34.11% (G2), who refused the procedure, were treated conventionally. There was no significant association between gender and the application of PRF (Chi-squared test, *p* = 0.171).

Among the patients on antiresorptive or antiangiogenic medication, 45.9% (44.64% in G1 and 48.3% in G2) underwent dental extractions, while 54.1% (55.36% in G1 and 51.7% in G2) had confirmed MRONJ and required other specific surgical interventions. The proportion of MRONJ cases was not significantly higher in either G1 (Chi-squared test, *p* = 0.750) or G2. The distribution between the two groups by pathology was roughly equal, despite the higher number of patients in G1 compared to G2. This allows for a comparison between the two groups in terms of healing rates, with or without PRF application, for both patients who required dental extractions and those already diagnosed with MRONJ who required other specific interventions.

In terms of location (upper or lower jaw), the two groups are similar, with no significant association between PRF application and the mandibular or maxillary location of osteonecrosis (Chi-squared test, *p* = 0.365). For the dental extractions performed, the distribution of teeth in the maxilla and mandible shows a slightly higher prevalence in the mandible, but this difference is not statistically significant (Mann–Whitney test, *p* = 0.581).

The average age of the patients was 64 years. The age differences between patients treated for MRONJ and those who underwent dental extractions were not significant (Mann–Whitney test, *p* = 0.930) (Table 1).

The percentage of patients on chronic oral anticoagulant medication was 16.47% of the total cases included in the study, while diabetic patients made up 23.53%. No correlation was observed between the associated conditions (diabetes or chronic anticoagulant treatment) and the occurrence of delayed healing or lack of healing, either in cases where PRF was applied or in cases treated with conventional surgical methods. There was no increased bleeding time mentioned in the patients’ reports.

From the perspective of the underlying conditions indicating the use of antiresorptive and antiangiogenic medication, the study included 20 patients treated for osteoporosis, 4 for multiple myeloma, and 61 patients with various forms of malignant tumors. Breast cancer with secondary metastases was the most common diagnosis in the study (34.12%), followed by prostate cancer (23.53%), osteoporosis (23.53%), kidney cancer (5.88%), multiple myeloma (4.7%), bronchopulmonary cancer (4.7%), and brain cancer (3.53%). The higher number of women included in the study is directly related to the general conditions requiring this type of medication, with 57.6% of the conditions being specific to females (breast cancer and osteoporosis) and only 23.53% to males (prostate cancer) (Table 2). The age differences based on the type of diagnosis that led to the initiation of antiresorptive therapy are not significant (Kruskal–Wallis test, *p* = 0.067). Additionally, the age differences related to treatment success and healing rates are also not significant (Kruskal–Wallis test, *p* = 0.284) (Table 3).

**Figure 1:**
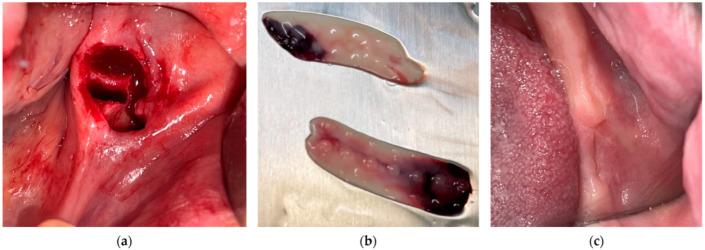
PRF application in post-extraction sockets: (**a**) post-extraction socket after wisdom tooth removal; (**b**) PRF membrane which was applied in the socket; (**c**) 4 weeks’ healing follow-up.

Of the total patients requiring dental extractions, 64.1% were treated with the application of PRF growth factors in the post-extraction sockets as shown in Figure 1, while 35.9% were treated without these growth factors.

**Figure 2:**
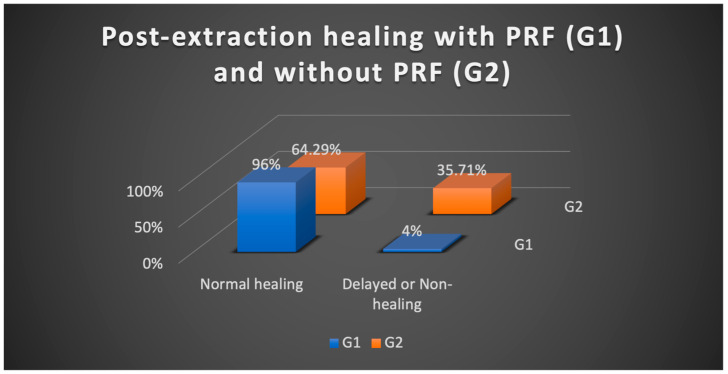
The percentage of normal healing cases depending on whether PRF was applied post-extraction or not.

A comparison between the two groups reveals a statistically significant association between the healing rate and the application of PRF in post-extraction sockets (Chi-squared test, *p* = 0.016). Specifically, in patients with high risk of MRONJ who underwent dental extractions, the proportion of non-healing and delayed healing cases is significantly higher among those without PRF (G2 = 35.71%) compared to the group with PRF (G1 = 4%), whereas the proportion of normal healing cases is significantly higher among those treated with PRF (G1 = 96%) than without it (G2 = 64.29%) (Table 4) (Figure 2).

In patients who underwent dental extractions, a detailed look at the non-healed and delayed healing cases is highlighted in Table 5. In Group 1, where PRF was used, 4% of patients experienced delayed healing within 4–8 weeks, with a late closure of the post-extraction dehiscence. However, by the 8-week follow-up, all patients had achieved good healing. In comparison, among patients who did not receive PRF, only 64.29% experienced normal healing, with none showing delayed healing, but 35.71% still had dehiscence at 8 weeks. The association between tooth extraction and the three types of healing at 8 weeks is significant (Chi-squared test, *p* = 0.005). In case of dental extractions in patients at high risk of developing MRONJ, there is only one case of delayed healing in the group where PRF was used, with no cases showing absence of healing. The patients in Group 2, where PRF was not used, 35.71% developed MRONJ, with five non-healing cases recorded (Table 5). Therefore, the proportion of non-healing cases was significantly higher in those without PRF (Chi-squared test, *p* = 0.007), while the proportion of normal healing was significantly higher in those with PRF (Chi-squared test, *p* = 0.03).

**Figure 3:**
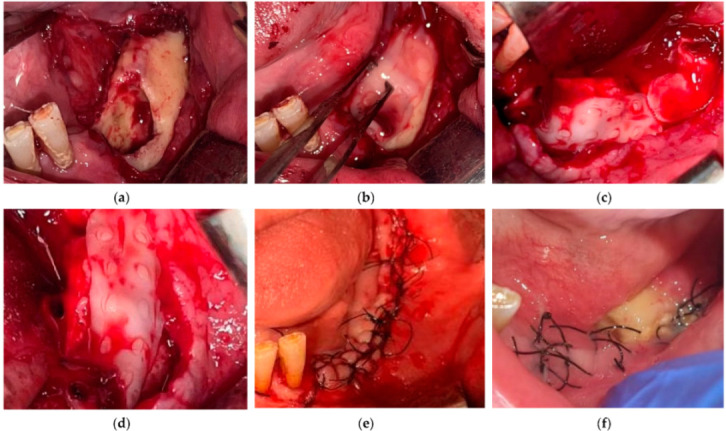
A non-healing case of MRONJ treatment using PRF: (**a**) bone defect after sequestrectomy; (**b**–**d**) PRF membrane application; (**e**) closure of the defect with a tension-free mucoperiosteal flap; (**f**) 2-week follow-up with delayed healing and bone exposure; at 8 weeks, the dehiscence was still there but without signs of local infection.

**Figure 4:**
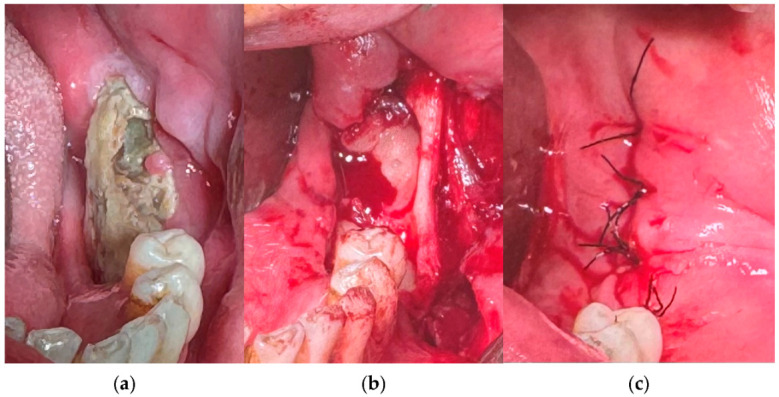
A normal healing case of MRONJ with PRF application: (**a**) mandibular bone sequestrum; (**b**) PRF application in the defect after curettage; (**c**) mucoperiosteal flap with hermetic suturing of the operated area.

In patients with confirmed MRONJ who were treated with conventional methods (G2) such as sequestrectomy, marginal refreshing, curettage, and defect reconstruction using adjuvant soft tissue flaps, the normal healing rate was 60%, similar to that of the patients who underwent dental extractions without PRF (64.29%). However, in Group 1 of the MRONJ patients, where PRF was applied to the defect after the removal of the bone sequestrum, as shown in Figure 3 and Figure 4, there was no significantly higher healing rate (77.42%) compared to those who did not receive PRF (60%). Therefore, in the case of MRONJ, it can be concluded that the association between PRF application and healing is not statistically significant (Chi-squared test, *p* = 0.299) (Table 4).

Regardless of the surgical intervention performed, both in patients at risk of developing MRONJ and in those with confirmed MRONJ, the cases in Group 1 where PRF was applied showed more instances of delayed healing than non-healing, compared to Group 2 patients, in whom non-healing cases predominated (Table 5).

## 4. Discussion

Following dental extractions or surgical procedures such as sequestrectomy and debridement in patients diagnosed with MRONJ, the outcome was assessed based on a classification of local healing type, in correlation with the official AAOMS case definition. In alignment with this, we considered as normal healing those cases where, at the 4-week follow-up, no local dehiscence, fistula, or local/regional infection was detected. Delayed healing was classified as cases where local dehiscence was present at the 4-week follow-up but had resolved with favorable local status by the 8-week follow-up. Patients who exhibited persistent soft tissue dehiscence or signs of local infection, with an oral or cutaneous fistula present at the 8-week follow-up, were classified as cases without healing [4].

The number of female patients included in the study (68.23%) was double compared to male patients, due to the inclusion of osteonecrosis cases specific to females in addition to cases with tumor-related pathology. In this study, no significant statistical correlations were observed between gender and healing for cases where PRF was applied. Similar studies also reveal a higher proportion of female patients, approximately 60–70% [16,17], without clinically significant correlation. In stage 0 of MRONJ cases, gender is not a relevant factor in the onset of this condition. However, gender seems to have a negative prognosis for male patients solely in advanced stages of MRONJ, likely due to men’s higher tolerance for pain, which can delay specialized treatment [18]. According to a meta-analytic review from 2023, the number of women on chronic antiresorptive and antiangiogenic medication is significantly higher than that of men, yet there remains an elevated risk among males for developing MRONJ as a complication following dento-alveolar surgical procedures [19].

The average age of the patients included in the study was 64 years, similar to other studies in the literature, where the age range varies between 58 and 70 years [16,20,21]. Neither in this study nor in other similar studies was an association observed between the patients’ age or tooth localization in maxilla or mandible and the risk of developing MRONJ following dental extractions [21,22].

In a study from 2023, in patients undergoing total knee arthroplasty, it was observed that concomitant administration of bisphosphonates and warfarin may increase post-surgical bleeding [23]. The presence of a correlation between the two types of treatment was followed in this study as well. The chronic anticoagulant treatment used by 16.47% of the patients simultaneously with the antiresorptive medication does not seem to have influenced, from any point of view, either the post-surgical bleeding times or the quality of healing.

Among the existing comorbidities of patients under chronic antiresorptive treatment, diabetes has the greatest clinical significance, uncontrolled blood sugar levels negatively influencing local healing [24]. However, neither in the present study nor in other similar studies was a correlation observed between healing and the presence of diabetes in relation to PRF use [22].

As of the last AAOMS Position Paper updated in 2022, the practice of a antiresorptive drug holiday still remained controversial. There are many studies on this subject with different conclusions, and a high level of evidence either for or against the use of a holiday has not been certified until now [4]. In the present study, the decision was made to interrupt chronic antiresorptive treatment three months prior to the surgical intervention and to resume it approximately one month after surgery. These time intervals were chosen in alignment with studies in the literature that demonstrate better local outcomes during the drug holiday period because of a reduction in osteoclastic inhibition that occurs after 3–4 months of treatment pause [25].

White blood cells combined with platelets and neutrophils within the PRF matrix are capable, by activating local angiogenesis mechanisms, of enhancing the healing of oral soft tissues. The blood components present in high concentrations in PRF membranes release chemotactic factors and growth factors that accelerate the process of local healing. Additionally, the fibrin loaded with platelet mass serves as a reservoir of growth factors, which are released gradually over a period of 10 to 14 days [26].

The first 7 days after dental extractions are critical for the subsequent healing of post-extraction sockets, particularly in patients undergoing chronic antiresorptive and antiangiogenic therapy, as these medications slow down the healing process of the gingival epithelium, inhibit the activity of osteoclasts, and accelerate the mechanism of apoptosis. This is the reason why the use of tissue growth factors such as PRF, applied to post-extraction sockets, can promote early epithelialization of the gingival wound and help prevent the occurrence of MRONJ [27,28].

In this prospective study, the post-extraction outcomes of a total of 105 teeth in 39 patients were monitored over the course of one year. In those cases where PRF was applied post-extraction, complete healing at 8 weeks was observed in all dental sockets, with delayed healing occurring in only one case at 4 weeks. In contrast, in the group of patients treated with conventional techniques without the use of PRF, only 64.29% of cases showed favorable outcomes. Approximately one-third of the patients from Group 2 (without PRF) experienced deficient post-extraction healing, with persistent dehiscence or alveolar fistulas observed at 4 and then 8 weeks. Therefore, in the present study, for dental extractions, the association between the use of PRF materials and local healing is significant (*p* = 0.005).

In a similar retrospective study from 2021 on the role of PRF in the prevention of MRONJ, 19.23% of the patients developed MRONJ in the control group, compared to the group in which PRF was applied, where no case of local dehiscence was observed [29]. Another study reveals a success rate of 100% in the group where PRF membranes were used after the dental extractions, while the classical technique achieved success in only 57% of cases [16]. Another type of study compared alveolar healing using a mucoperiosteal flap against the application of PRF with simple marginal closure. The use of PRF demonstrated greater efficacy, even when compared to primary closure techniques for the alveolar defect. It offered clear advantages, both in terms of its minimally invasive nature and its rapid technique [30,31,32]. Numerous similar studies in the literature reveal a statistically significant difference between study groups where PRF was applied and control groups, whose patients did not benefit from tissue regeneration materials [33]. The encouraging results of the present study support the current research directions, increasingly favoring the use of tissue regeneration materials such as PRF in the post-extractional socket to prevent the occurrence of MRONJ in patients undergoing chronic antiangiogenic and antiresorptive medication.

In cases of confirmed MRONJ, the use of PRF does not appear to yield results as favorable as the application of PRF in post-extraction sockets. When analyzing the progression of osteonecrosis cases in patients treated with PRF compared to traditional treatment methods, a slight improvement in local healing has been observed, with the percentage of normal healing cases increased from 60% to 77.42%. However, this improvement lacks sufficient statistical significance (*p* = 0.299) to conclusively determine the clear benefit of using PRF in the progression of MRONJ cases within the selected patient sample in this study.

Regarding the effects of PRF application in already diagnosed cases of MRONJ, the studies in the literature are controversial. Various studies in the literature reveal a favorable impact of PRF application following alveolar curettage in patients diagnosed with different stages of MRONJ. However, drawing statistically significant conclusions remains challenging, as these studies are either case reports, involve small patient groups, or present significant heterogeneity regarding patient characteristics and perioperative management [16,34,35]. A review by Bracher A.I. et al. in 2021 identified only one randomized controlled trial, which concluded that the use of PRF demonstrates only modest superiority by improving infection rates and reducing pain [17]. An improvement in local healing of patients with confirmed MRONJ following PRF application is also observed in the present study, with an increase in the proportion of normal healing cases from 60% to 77.42%; however, this difference lacks statistical significance when compared to the evident importance of PRF use in post-extraction sockets.

In a systematic review including 30 articles, Nowak S.M et al. assessed the effectiveness of using autologous platelet concentrates (APCs) in the prevention and treatment of patients diagnosed with MRONJ. Application of tissue growth factors of the APC type has been shown to be useful in preventing the development of MRONJ cases (*p* < 0.001), but in the treatment of existing MRONJ, it does not appear to have a statistically significant result (*p* = 0.08) [36]. These conclusions are in line with the results obtained in our clinical study.

Based on the statistical analyses conducted in the present study and correlating them with similar studies from the literature, the effectiveness of PRF in the treatment of already confirmed MRONJ remains uncertain. On the other hand, PRF proves to have an adjuvant role in the prevention of MRONJ development in patients requiring tooth extractions.

This study is limited by the small sample size and the lack of long-term follow-up to confirm the absence of local recurrences over time. Further randomized controlled studies are necessary, involving larger patient samples with minimal variations in underlying systemic conditions and types of antiresorptive treatment administered (intravenous versus oral).

## 5. Conclusions

The use of PRF in patients undergoing chronic antiresorptive and antiangiogenic treatment improves local post-extraction healing compared to cases where only traditional surgical methods are used. In contrast, in confirmed MRONJ cases, the use of PRF does not provide statistically significant benefits.

Application of PRF in post-extraction sockets of patients on chronic antiresorptive medication to prevent MRONJ occurrence has shown a statistically significant increase in efficacy (*p* = 0.03) compared to the group of patients who did not receive PRF. The percentage of patients who achieved normal healing at 4 and 8 weeks was 96% and subsequently 100% with PRF application, compared to only 64.29% without PRF use.

The same cannot be said for patients who presented for the treatment of already existing MRONJ. In this group, the use of PRF provided a slight benefit in the percentage of cases with normal healing, with an increase from 60% to 77.42%, but without statistical significance.

The present study has certain limitations related to the small sample size and the relatively short follow-up period of only 8 weeks, which assesses only adequate soft tissue healing without monitoring the long-term evolution of the osteonecrotic bone lesions. Future large-scale, randomized prospective clinical studies are necessary to clearly evaluate the effectiveness of PRF use in the management and treatment of MRONJ.

## Figures and Tables

**Table 1:** Age distribution and difference among the patients who underwent surgery for pre-existing MRONJ and those at high risk of developing MRONJ who underwent dental extractions.

AGE	MRONJ (*n* = 46)	Tooth Extraction (*n* = 39)	*p*-Value
Mean ± SD	64.63 ± 9.86	64.28 ± 8.78	0.930
Median (Q1–Q3)	68 (55.25–73)	64 (58–73.5)	

MRONJ—total number of patients with pre-existing MRONJ who underwent sequestrectomies and local debridement; Tooth Extraction—total number of patients with high risk of MRONJ who underwent dental extractions; SD—standard deviation; Q1,3—the first, third quartile; the Mann–Whitney U test was used to calculate *p*-value.

**Table 2:** Clinical characteristics: underlying disease that required antiresorptive and antiangiogenic therapy and age distribution (SD—standard deviation; SE—standard error of mean).

Malignancies	N	Mean of Age	SD	SE	Coefficient of Variation
Brain cancer	3	69	2.646	1.528	0.038
Breast cancer	29	61.793	10.662	1.98	0.173
Pulmonary cancer	4	60.5	5.196	2.598	0.086
Kidney cancer	5	56.4	4.336	1.939	0.077
Multiploe myeloma	4	65.5	11	5.5	0.168
Osteoporosis	20	67.15	8.816	1.971	0.131
Prostate cancer	20	67.6	7.632	1.707	0.113

**Table 3:** The correlation between patients’ age and treatment success.

AGE	Delayed Healing (*n* = 6)	Non-Healing (*n* = 13)	Normal Healing (*n* = 66)	*p*-Value
Mean ± SD	62.50 ± 10.60	61.08 ± 11.72	65.32 ± 8.66	0.284
Median (Q1–Q3)	61.5 (54.5–70)	59 (53–74)	68 (59–73)	

SD–standard deviation; Q1,3–the first, third quartile; the Kruskal–Wallis test was used to calculate *p*-value.

**Table 4:** Normal healing versus delayed/non-healing cases depending on the type of diagnosis (extraction or MRONJ) and the study group.

Diagnosis		Healing		*p*-Value
		Delayed/Non	Normal	
MRONJ *n* (%)	G1 PRF	7 (22.58%)	24 (77.42%)	0.299
	G2 Non-PRF	6 (40.00%)	9 (60.00%)	
Tooth extraction *n* (%)	G1 PRF	1 (4.00%)	24 (96.00%)	0.016 *
	G2 Non-PRF	5 (35.71%)	9 (64.29%)	

MRONJ—patients with pre-existing MRONJ who underwent sequestrectomies and local debridement; Tooth extraction—patients with high risk of MRONJ who underwent dental extractions; *—significant difference according to the Chi-squared test.

**Table 5:** Healing outcome at 4 and 8 weeks after tooth extractions depending on PRF use.

Diagnosis		8 Weeks Follow Up			*p*-Value
		Delayed Healing	Non-Healing	Normal Healing	
MRONJ *n* (%)	G1-PRF	4 (12.90%)	3 (9.68%)	24 (77.42%)	0.133
	G2 Non-PRF	1 (6.67%)	5 (33.33%)	9 (60.00%)	
Tooth extraction *n* (%)	G1-PRF	1 (4.00%)	0 (0.00%)	24 (96.00%)	0.005 *
	G2 Non-PRF	0 (0.00%)	5 (35.71%)	9 (64.29%)	

*—significant difference according to the Chi-squared test.

## Data Availability

The data presented in this study are available on request from the corresponding author. The data are not publicly available due to restrictions related to the privacy of the patients and the funding protocol.
